# Systematic Analysis of Cell Cycle Effects of Common Drugs Leads to the Discovery of a Suppressive Interaction between Gemfibrozil and Fluoxetine

**DOI:** 10.1371/journal.pone.0036503

**Published:** 2012-05-02

**Authors:** Scott A. Hoose, Camille Duran, Indranil Malik, Shabnam Eslamfam, Samantha C. Shasserre, S. Sabina Downing, Evelyn M. Hoover, Katherine E. Dowd, Roger Smith, Michael Polymenis

**Affiliations:** 1 Department of Biochemistry and Biophysics, Texas A&M University, College Station, Texas, United States of America; 2 Department of Veterinary Pathobiology, Texas A&M University, College Station, Texas, United States of America; University of Minnesota, United States of America

## Abstract

Screening chemical libraries to identify compounds that affect overall cell proliferation is common. However, in most cases, it is not known whether the compounds tested alter the timing of particular cell cycle transitions. Here, we evaluated an FDA-approved drug library to identify pharmaceuticals that alter cell cycle progression in yeast, using DNA content measurements by flow cytometry. This approach revealed strong cell cycle effects of several commonly used pharmaceuticals. We show that the antilipemic gemfibrozil delays initiation of DNA replication, while cells treated with the antidepressant fluoxetine severely delay progression through mitosis. Based on their effects on cell cycle progression, we also examined cell proliferation in the presence of both compounds. We discovered a strong suppressive interaction between gemfibrozil and fluoxetine. Combinations of interest among diverse pharmaceuticals are difficult to identify, due to the daunting number of possible combinations that must be evaluated. The novel interaction between gemfibrozil and fluoxetine suggests that identifying and combining drugs that show cell cycle effects might streamline identification of drug combinations with a pronounced impact on cell proliferation.

## Introduction

Adjusting rates of cell proliferation is the objective of many therapeutic strategies. Most often, the goal is to impede or block cell proliferation of target cells, as with chemotherapy in cancer. In other cases, as in tissue regeneration, the goal is to promote cell proliferation. Proliferating eukaryotic cells pass through a series of highly regulated cell cycle phases, culminating with mitosis [1]. Hence, drugs that influence the timing of cell cycle transitions are useful in efforts to adjust rates of cell proliferation.

Identifying drugs that potentiate the effects of other drugs is the leading therapeutic strategy in the treatment of numerous diseases, such as cancer [2], tuberculosis [3] and HIV-AIDS [4]. Conversely, drug interactions may suppress a desired response, or even lead to a harmful outcome. Screening libraries composed of a few hundred thousand compounds for a sought-after effect of a single chemical is now common [5]. However, testing all the possible combinations, even binary ones, of these chemicals represents a formidable obstacle [6].

Here we report a systematic analysis of cell cycle progression of yeast cells exposed to a panel of FDA-approved drugs. We document novel cell cycle effects of several compounds. We also reasoned that drugs that affect cell cycle progression might be more likely to display interactions with other such drugs, and thereby greatly impact overall cell proliferation. We demonstrate one such novel drug interaction, between gemfibrozil and fluoxetine.

## Results and Discussion

**Figure 1:**
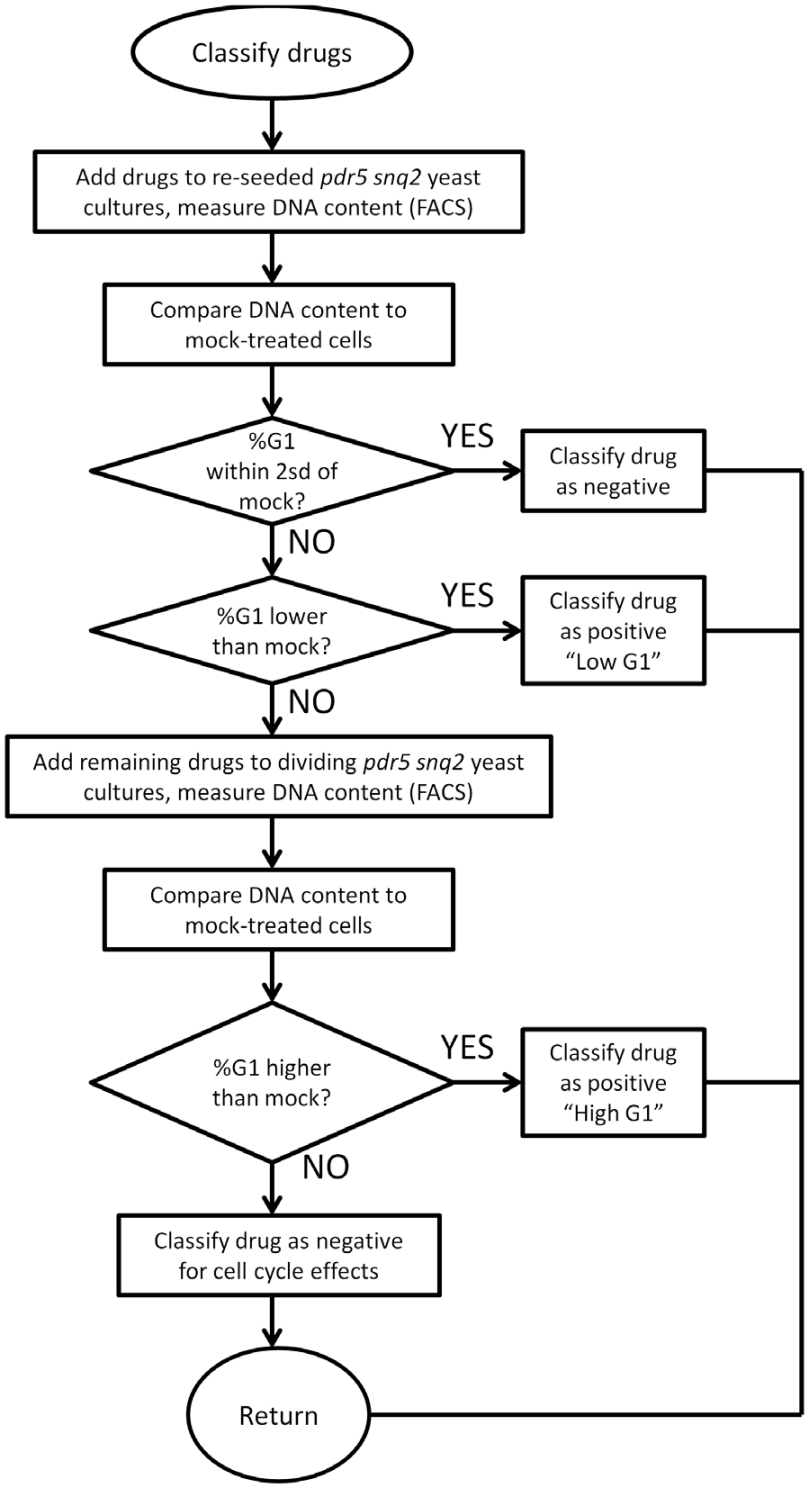
Decision flow-chart diagram of our primary analysis. This diagram summarizes our DNA content measurements using the *pdr5Δ, snq2Δ* strain. See text for details.

**Figure 2:**
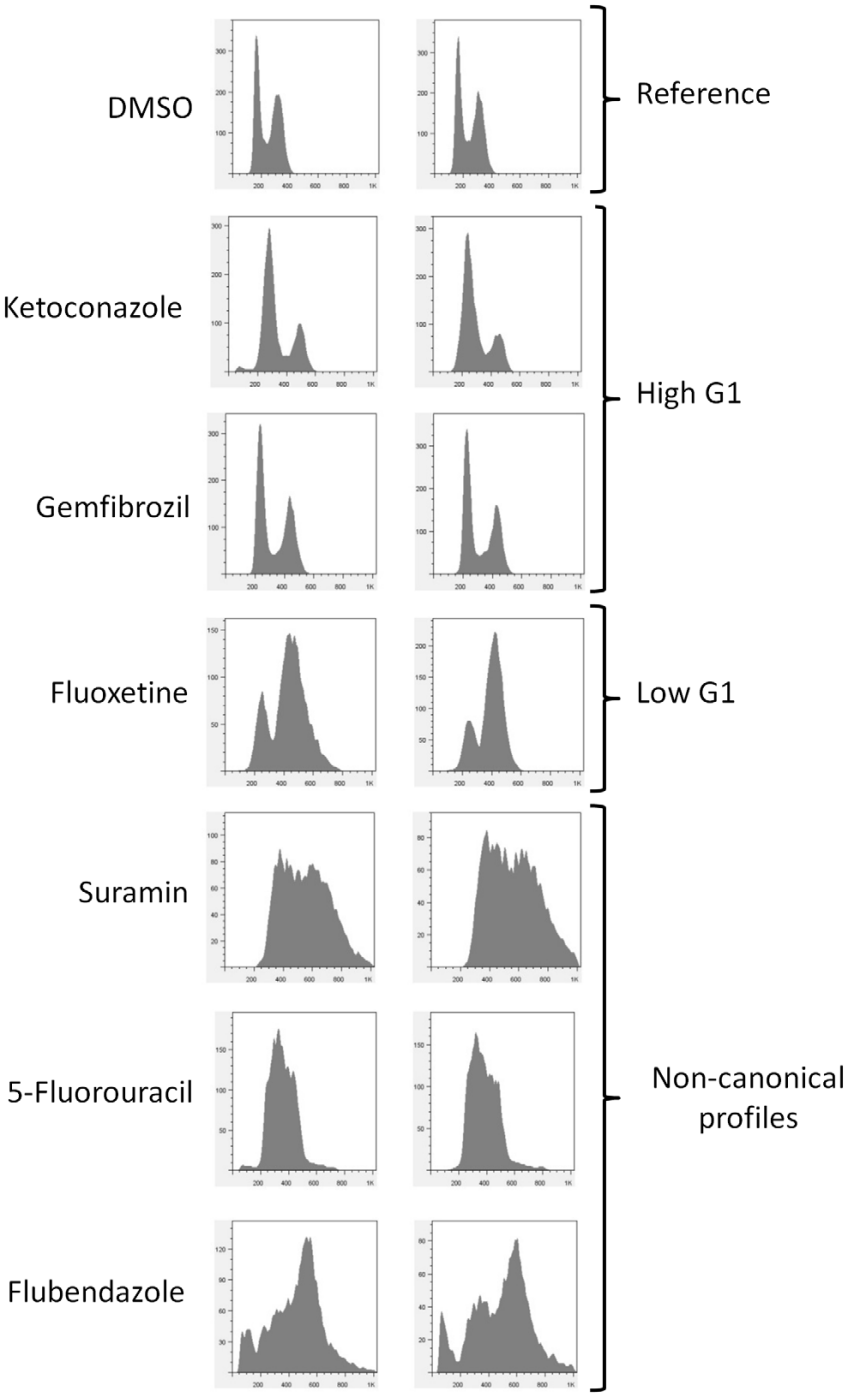
Representative DNA content histograms. Independent experiments of the indicated samples are shown in each case. Fluorescence is plotted on the x-axis, while the number of cells analyzed is on the y-axis. Reference samples were treated with DMSO, shown at the top. Examples of “High G1” profiles include cells treated with ketoconazole or gemfibrozil, while cells treated with fluoxetine give rise to a “Low G1” DNA content profile. At the bottom, we show a few examples of complex DNA content histograms that were unquantifiable. These include profiles of cells treated with suramin and 5-fluorouracil (antineoplastic agents), and flubendazole (a microtubule blocker used as anti-nematodal).

We used a commercially available panel of 640 FDA-approved drugs (see Materials and Methods). The target cells were *Saccharomyces cerevisiae* budding yeast, a model system of eukaryotic cell cycle studies [1]. We monitored the effects of each drug on cell cycle progression by measuring the DNA content of the cells by flow cytometry [7] (see Figure 1, and Materials and Methods). The G1 phase of any given cell cycle lasts from the end of the previous mitosis (M phase) until the beginning of DNA synthesis (S phase). Any drug that alters the length of the G1 phase relative to the rest of the phases of the cell cycle will alter the DNA content profile. We quantified each sample in an automated manner, recording the percentage of cells with unreplicated genome (%G1, see Materials and Methods). We did not quantify complex profiles (see Figure 2), and we excluded these drugs from further analyses. At the beginning and end of most batches of samples, we measured the reference sample (a yeast strain that lacks the multidrug transporters Pdr5p and Snq2p, mock-treated with DMSO; see Materials and Methods), which was cultured and processed along with the cultures that were treated with drugs. We evaluated each drug in at least two independent experiments. We deposited all raw flow cytometry data in a public database (see Dataset S1, and Materials and Methods).

**Figure 3:**
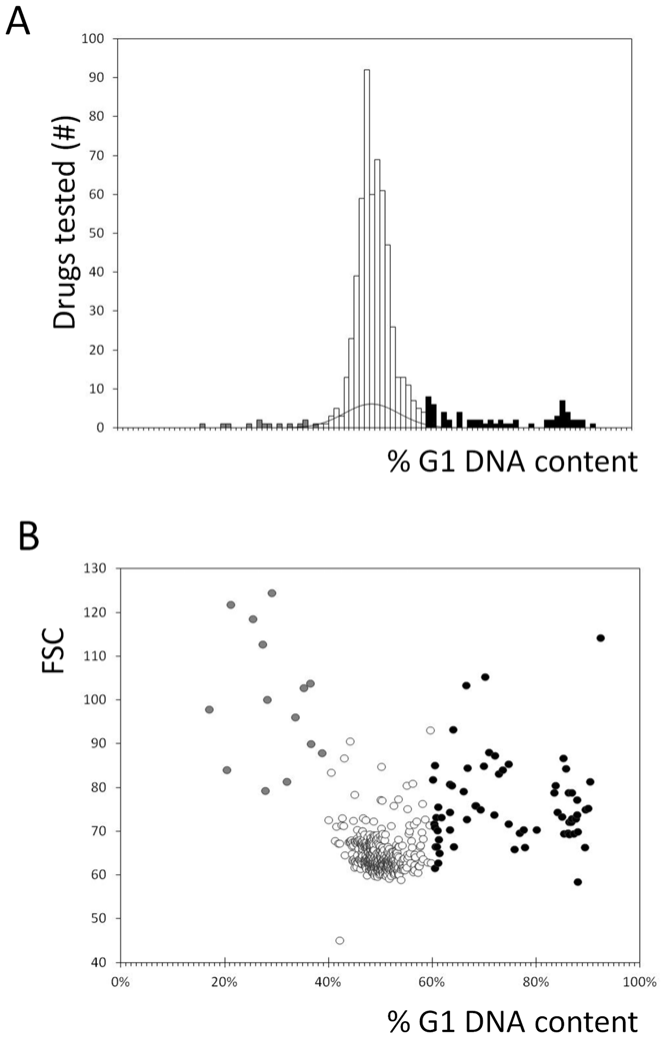
DNA content analysis identifies drug effects on cell cycle progression. A, Cumulative histogram displaying the percentage of cells in the G1 phase of the cell cycle (%G1), for cells treated with a panel of FDA-approved drugs. The bin width of the histogram is 1%, with each bin containing all the drugs with values within the bin boundaries. The black line superimposed to this histogram is the normal distribution fit of the %G1 values of the reference sample. Bins with values >2 sd from the mean of the wild type distribution are in grey (“Low G1” group) and black (“High G1” group). B, From all the samples we analyzed by flow cytometry, the %G1 is on the x-axis, and the forward angle scattering (FSC) values on the y-axis. We colored the data points of the sub-groups as in A.

To identify drugs that altered the cell cycle, we compared the frequency distribution of cultures treated with drugs against a normal distribution fit of the reference (n = 82) samples (Figure 3A). Samples that had a %G1 greater or less than two standard deviations from the mean of the reference sample distribution were considered to differ significantly from the mock-treated samples (Figures 1 and 3A). Drugs that led to an increase (%G1>60.00%) in the percentage of cells with unreplicated DNA formed the “High G1” group, while others led to a mitotic delay and a “Low G1” (%G1<38.76) DNA content (see Figure 3A, and Dataset S1). In this initial screen, we added the drugs to cultures diluted from an overnight stationary phase culture, where most cells would be in the G1 phase of the cell cycle [1]. Hence, drugs in samples with a “High G1” DNA content may have arrested cell cycle progression non-specifically. In that case, the high G1 DNA content reflected the state of the starting culture, and not cell cycle effects of the drugs. To exclude such possibilities, we re-tested the “High G1” drugs by adding them to actively dividing cells (see Figure 1). Overall, from this primary analysis we identified 27 compounds that interfered with progression in the G1 phase of the cell cycle, before initiation of DNA replication, resulting in a “High G1” DNA content (see Table S1). Another 12 drugs affected mitotic progression, resulting in a “Low G1” DNA content (see Table S2).

Along with DNA content, we also analyzed the forward scatter (FSC) from the same flow cytometry experiments (see Figure 3B). FSC values often serve as a proxy for cell size, but they are also affected by cell shape and intracellular composition [8]. We noticed that most drugs in the “Low G1” group had elevated FSC values compared to the group with no cell cycle effects (Figure 3B). This is consistent with the notion that mitotic delay leads to an increase of cell size. It should also be noted that yeast cells in mitotic phases of the cell cycle are budded [1]. Hence, their irregular shape may also contribute to an increase in FSC values. An increase of FSC values was also evident for a significant fraction, but not all, of drugs in the “High G1” group (Figure 3B).

We are not aware of other systematic studies of drug effects on cell cycle progression measured by DNA content analyses. Our results reveal that several drugs currently and commonly used for human therapy have specific effects on the eukaryotic cell cycle. The higher number of drugs that interfered with G1 progression likely reflects the fact that cells commit to initiation of cell division in the G1 phase [1], [9], [10]. Among the “High G1” group, we noted antifungals that inhibit biosynthesis of ergosterol, a component of fungal membranes [11], and rapamycin, a potent inhibitor of the TOR pathway known to block G1 progression [12]. Overall, however, there was a diverse range of compounds in the “High G1” group (see Table S1). Although most drugs in the “Low G1” group have well established mitotic roles (see Table S2), we noted that the highest-ranked drug from this group was fluoxetine (brand name Prozac). To our knowledge, this is the first time that such strong cell cycle effects have been reported for fluoxetine.

Since we did our primary analysis in a sensitized *pdr5Δ*, *snq2Δ* yeast strain, we then tested the drugs that led to the “High G1” and “Low G1” groups against the *PDR5^+^, SNQ2^+^* wild type reference strain BY4741. We found that several drugs were not effective in this case. For example, lovastatin, which leads to a G1 arrest in mammalian cells [13], had no effect in *PDR5^+^, SNQ2^+^* yeast cells (see Table S1). This is consistent with an earlier report that yeast cells are sensitive to lovastatin in a *pdr5Δ* -dependent manner [14]. Nonetheless, about half of the drugs in both groups remained effective in cells with intact multidrug transporters (see Tables S1 and S2).

Among drugs that led to a “High G1” DNA content, we further examined the cell cycle effects of the potent antilipemic gemfibrozil [15], a Peroxisome Proliferator-Activated Receptor α (PPARα) agonist. To our knowledge, a G1 cell cycle role for gemfibrozil has not been reported, in any system. The High G1 DNA content could result from roles specific to G1 progression, or manifest in G1 as a “carryover” from roles in other cell cycle phases. To distinguish between these two possibilities, we added gemfibrozil to highly synchronous newborn G1 cells, obtained by centrifugal elutriation [16], [17].

**Figure 4:**
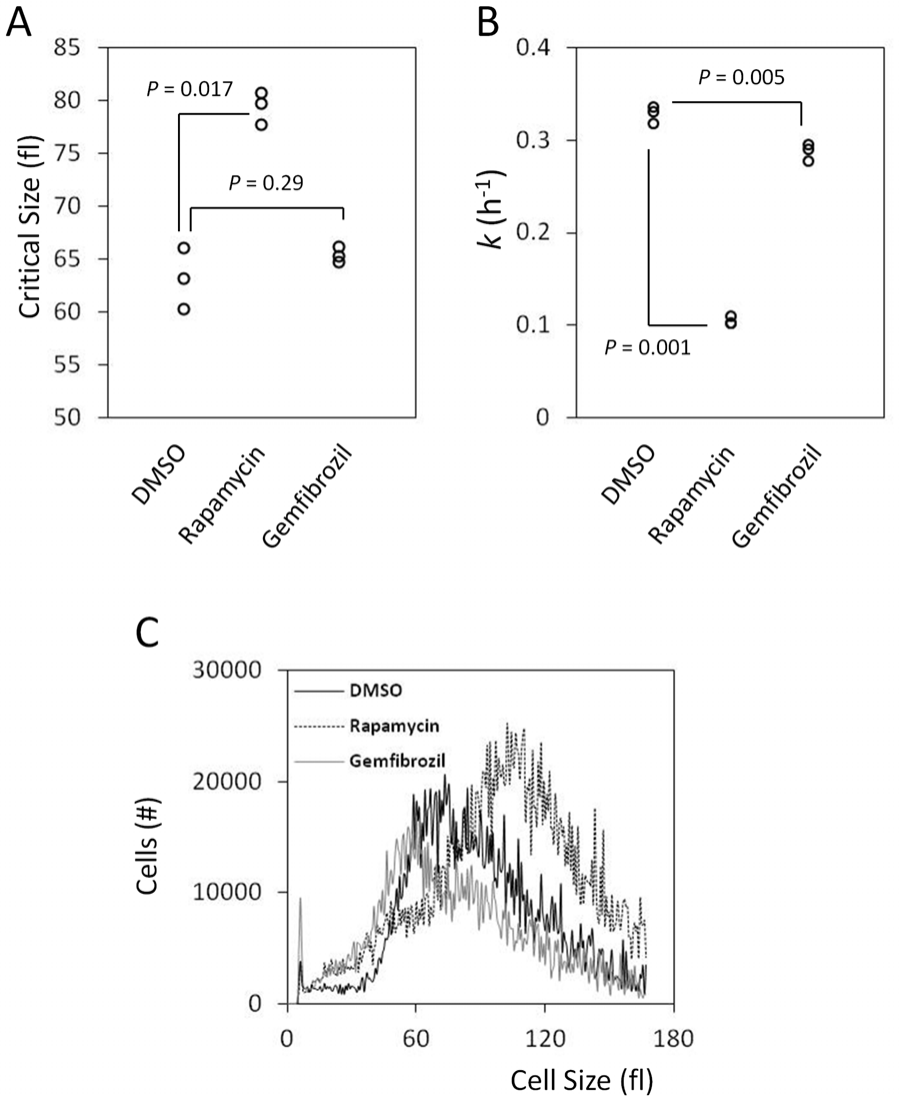
Gemfibrozil delays initiation of DNA replication. A, The critical cell size (shown in fl) of diploid BY4743 cells treated with DMSO, rapamycin (0.1 µg/ml) or gemfibrozil (50 µg/ml), was measured from synchronous elutriated cultures, in YPD medium. The data points shown were from three independent experiments in each case. The *P* values shown were calculated from paired, two-tailed *t* tests, assuming unequal variance. The data used to calculate these parameters are shown in Figure S1. B, The specific rate of cell size increase constant *k* (in h^−1^) was measured from the same elutriation experiments shown in a, assuming exponential growth. The data used to calculate these parameters are shown in Figure S1. C, The cell size distributions of the indicated cell populations, proliferating asynchronously in YPD medium, were measured using a channelyzer (see Materials and Methods, and [22]). Cell numbers are plotted on the y-axis and cell size (in fl) on the x-axis. Daughter “birth” size was defined as the maximum size of the smallest 10% of cells on the left side of the cell size distribution of each sample [22].

As a function of time, we then measured cell size and the percentage of budded cells (budding correlates with initiation of DNA replication in yeast [1]). This allowed us to measure the length of the G1 phase accurately, by calculating two parameters: i) the “critical size” these newborn daughter cells must attain to initiate cell division; ii) the rate (“growth rate”) at which they grow to their critical size. DMSO-treated cells had a critical size of 63.2±2.4 fl and a specific growth rate constant *k* = 0.328±0.008 h^−1^ (Figure 4). Rapamycin markedly prolonged the G1 phase, because cells had to reach a substantially larger critical size (79.4±1.2 fl) before they could initiate DNA replication (Figure 4A). Rapamycin-treated cells also grew very slowly (*k* = 0.104±0.004 h^−1^, Figure 4B), although this effect was evident ∼1 h after addition of the drug (Figure S1). We found that cells treated with gemfibrozil delayed initiation of DNA replication, not because they had altered critical size (65.4±0.6 fl, Figure 4A), but because they reached that size slower than cells treated with DMSO did (*k* = 0.287±0.07 h^−1^, *P* = 0.005, Figure 4B). In addition, from the cell size distributions of asynchronously dividing cells, we obtained the “birth size” of newborn cells (see Materials and Methods). While DMSO-treated cells had a “birth size” of 40.3±2.7 fl under these growth conditions, gemfibrozil-treated newborn cells were significantly smaller (30.1±4.7 fl, *P* = 0.04, Figure 4C). Taken together, these data show that the smaller “birth size” and slower “growth rate” of cells treated with gemfibrozil lengthen the G1 phase.

**Figure 5:**
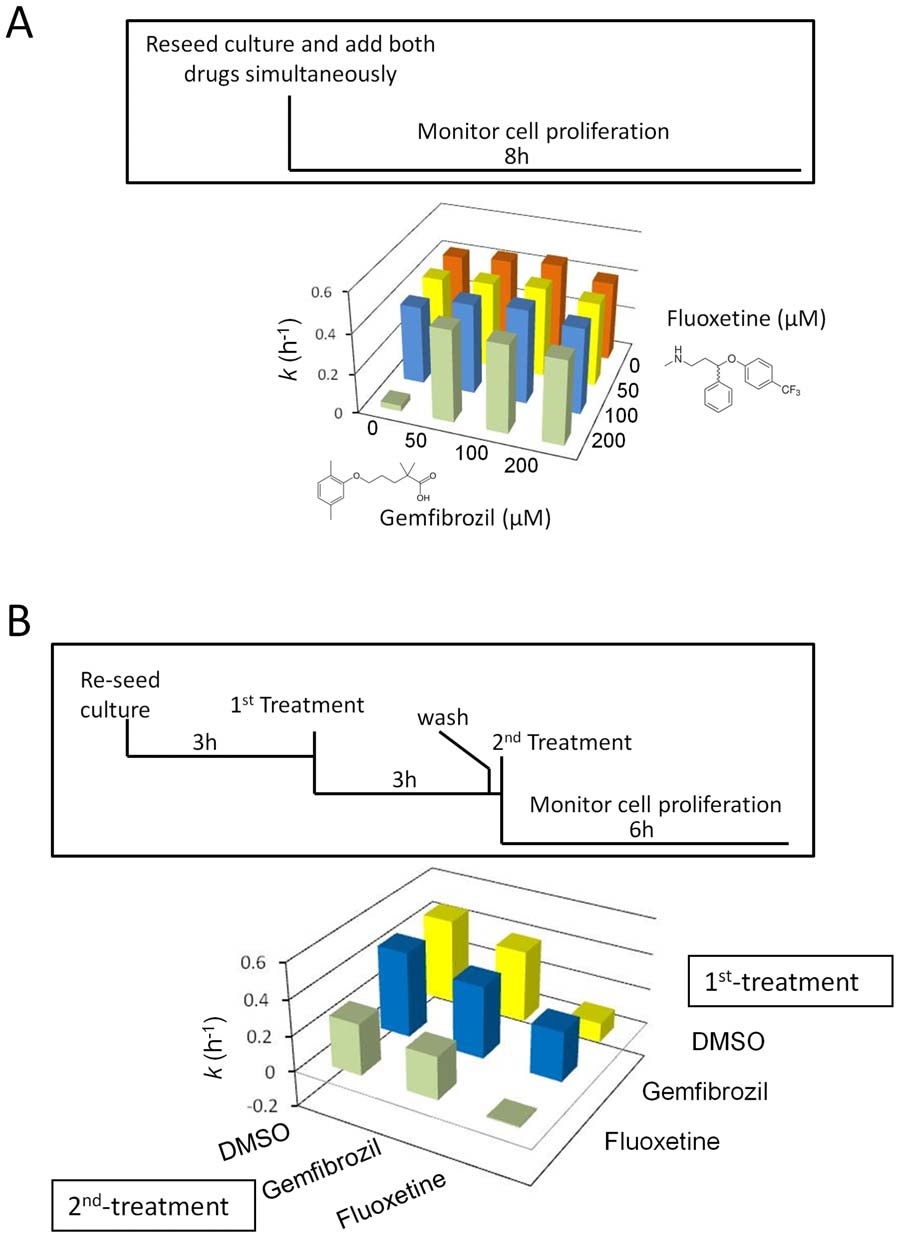
A novel interaction between gemfibrozil and fluoxetine. A, Fluoxetine strongly inhibits yeast cell proliferation, but it is suppressed by gemfibrozil. We added to freshly reseeded wild type haploid yeast (BY4741) cells DMSO, fluoxetine and gemfibrozil at the binary combinations and concentrations shown. We then monitored cell proliferation hourly, for 8 h (see Materials and Methods). The specific growth rate constant (*k*) for each combination is shown. The errors associated with these measurements are shown in Table S2. B, DMSO, fluoxetine and gemfibrozil were added to dividing cells at 200 µM in binary combinations, sequentially, in the order shown. Cell proliferation was monitored for 6 h as in a, with the specific growth rate constant (*k*) for each combination shown. Data from one representative experiment is shown. Suppressive effects of gemfibrozil on fluoxetine arising from order of addition were assessed by calculating growth rate constant (*k*) folds for gemfibrozil treatment over DMSO control for all experiments, initial treatment with gemfibrozil yielding a fold of 2.51 +/− 0.25, versus final treatment, 0.71 +/− 0.21, P-value = 0.000146.

Next, we focused on the effects of gemfibrozil and fluoxetine on overall cell proliferation rates. We tested these drugs alone and in combination, at several doses (Figure 5A). We found that gemfibrozil did not significantly affect overall cell proliferation at the doses tested (Figure 5). Hence, the prolongation of the G1 phase by gemfibrozil is likely accompanied by compensatory shortening of subsequent cell cycle phases, resulting in similar overall generation time. On the other hand, fluoxetine arrested proliferation of yeast cells at 200 µM (Figure 5A, first green bar to the left; and Table S3, bottom left cell). To our knowledge, the near complete inhibition of yeast cell proliferation by fluoxetine has not been reported. Remarkably, however, addition of gemfibrozil even at a 4-fold less molar concentration fully suppressed the inhibitory effects of fluoxetine (see Figure 5A, compare the left green bar to the other green bars; and Table S3, last row).

We then added the two drugs not simultaneously, but in different order, removing the first drug before adding the second (Figure 5B). We found that gemfibrozil suppressed fluoxetine's anti-proliferative effects only if added before (representative experiment in Figure 5B, compare the blue and yellow bars on the right; and Table S4, compare the top and middle cells in the 3^rd^ column), but not after fluoxetine (Figure 5B, compare the left and middle green bars; and Table S4, compare the left and middle cells in the 3^rd^ row). These results suggest that the suppressive interaction between gemfibrozil and fluoxetine is not due to extracellular interaction or competition for transport between the two drugs. Furthermore, the results from the order-of addition experiment suggest that gemfibrozil acts upstream, since it does not reverse fluoxetine's inhibition of cell proliferation. Instead, it appears that fluoxetine cannot inhibit cell proliferation in the context of gemfibrozil's prior action.

Understanding the basis of the interaction between gemfibrozil and fluoxetine requires a mechanistic understanding of their function in yeast cells. We examined the combined effects on cell proliferation between gemfibrozil and fluoxetine because of the novel cell cycle effects of each compound, affecting different phases of the cell cycle. We would like to note, however, that the suppressive interaction between the two compounds could be unrelated to their cell cycle effects. For example, gemfibrozil might induce expression of proteins that do not interfere with cell cycle progression, but may cause fluoxetine resistance. Fluoxetine is an anti-depressant thought to act as a serotonin-specific reuptake inhibitor [18]. Hence, the effects we described for fluoxetine in yeast appear to result from some other mechanism. Similarly, nuclear receptors of the PPARα/RXR type, the target of gemfibrozil, are thought to be unique to animals and sponges [19], [20], but ancestral analogs may exist in yeast [21]. Nonetheless, although the effects of fluoxetine and gemfibrozil on yeast cells we described above likely represent off-target modes of action, they may act similarly in other eukaryotic organisms, including humans. In conclusion, our results suggest that monitoring the effects of drugs on cell cycle progression reveals unexpected cellular roles of widely prescribed compounds. Finally, although we did not test all possible combinations of the compounds that affected cell cycle progression, at least in the case of gemfibrozil and fluoxetine, our results suggest that combining such compounds may also be an effective strategy to identify novel drug interactions.

## Materials and Methods

### Yeast strains

For our primary analysis, we used the *S. cerevisiae* strain JTY2953 (MATa *pdr5::TRP1 snq2::hisG ade2-101 his3-Δ200 leu2-Δ1 lys2-801am trp1-Δ63 ura3–5*2; a generous gift from Dr. Paul deFigueiredo, Texas A&M University). For the elutriation experiments in Figure 4 we used the diploid strain BY4743 (MATa/α *his3Δ1/his3Δ1 leu2Δ0/leu2Δ0 lys2Δ0/LYS2 MET15/met15Δ0 ura3Δ0*/ura3Δ0; commercially available from Open Biosystems). For all other experiments, we used the haploid strain BY4741 (MATa *his3Δ1 leu2Δ0 met15Δ0 ura3Δ0*; commercially available from Open Biosystems).

### Media and culture conditions

In all experiments, strains were cultured at 30°C in YPD (1% yeast extract, 2% peptone, 2% dextrose). For our primary analysis with the JTY2953 strain, overnight cultures were diluted 1∶200 and aliquoted into 96-well plates, 198 µl per well. To each well we then added 2 µl of a drug stock solution (2 mg/ml in DMSO), resulting in a final drug concentration of 20 µg/ml. At the four corner wells of each 96-well plate, the cultures were treated with DMSO only. These cultures served as the mock-treated reference samples. The plates were then placed at 30°C and incubated for 6–7 h. Each of the 200 µl cultures were then transferred to microcentrifuge tubes containing 500 µl ethanol, and sonicated for 5 s. For the experiments where the drugs were added in dividing JTY2953 cells, the overnight cultures were diluted 1∶400 and incubated for 3 h at 30°C. We then added the drugs of interest and incubated the plates at 30°C for another 6 h before fixing the samples in ethanol. For DNA content measurements in BY4741 cells, which proliferate faster than JTY2953 cells do, overnight cultures were diluted 1∶400, cultured for 2.16 h before we added the drugs of interest, and then cultured for another 4.33 h before they were fixed in ethanol.

### Cell size determinations

To obtain size distributions from asynchronous cultures, overnight cultures of BY4743 cells were diluted 1∶500 in fresh medium, and incubated for 2 h at 30°C. We then added the drugs of interest and incubated at 30°C for another 4 h. Cell size was then measured with a Beckman Z2 Channelyzer. For each sample we analyzed, we obtained size distributions from two different dilutions of cells. The average of the geometric mean of each size distribution was recorded. We used the Accucomp Beckman software package to obtain the statistics of each size distribution.

### Measurements of critical size and growth rate from elutriated cultures

For isolation of early G1 daughter cells, cultures were grown in YPD at 30°C to a density of ∼1–5×10^7^ cells/ml, then fractionated with a Beckman JE-5.0 elutriator as described previously [16]. Early fractions containing predominantly (>95%) small unbudded cells were collected by centrifugation, re-suspended in fresh medium and aliquoted in three separate flasks. To each flask, we then added as indicated rapamycin (at 0.1 µg/ml), gemfibrozil (at 50 µg/ml), or DMSO alone. After testing several doses of each drug and measuring the DNA content, we decided to use these concentrations because they were the lowest ones that resulted in consistently pronounced effects in this strain background. The cultures were incubated at 30°C. Every 20 min we monitored the percentage of budded cells and cell size. The “critical size” is the size at which 50% of the cells have budded in these experiments, and it was calculated as we described elsewhere [16]. To calculate “growth rate” assuming exponential growth, we plotted the natural log (ln) of cell size as a function of time (in h), see Figure S1. We fit the data to a straight line using the regression function in Microsoft Excel. From the slope of the line, we obtained the specific rate of cell size increase constant (*k*, in h^−1^). The average of all experiments (n = 3) for each treatment was then calculated, along with the associated standard deviation.

### Staining for DNA content analyses

Fixed cells were stored at 4°C overnight to 14 days. Cells were collected by centrifugation and stained overnight in 0.5 ml staining solution containing 50 mM sodium citrate pH 7.0, 0.25 mg/ml RNaseA, and 1 µM SYTOX Green (Molecular Probes). Samples were stored at 4°C overnight in opaque containers. Cell suspensions were sonicated briefly at the fixing and staining steps and immediately before flow cytometry.

### Flow cytometry data acquisition, deposition and analysis

Stained cells were analyzed on a FACSCalibur (Becton Dickinson Immunocytometry Systems) flow cytometer, using CellQuest (version 3.3; Becton Dickinson Immunocytometry Systems) acquisition software. Sytox Green fluorescence was collected through a 515/30-nm bandpass filter, and list mode data were acquired for 10,000 cells defined by a dot plot of FSC versus SSC. Prior to each experiment, standard beads (Cyto-Cal Multifluor Intensity Beads, Thermo Scientific) were used to calibrate the flow cytometer, and photomultiplier tube voltages were adjusted to place the highest intensity bead in the same channel (+/− 3). FACS files were archived at *Cytobank*. Automated quantification of the DNA content histograms was done with FlowJo 7.5 software. To exclude particulate non-yeast events, which had both very low forward scatter (FSC) and low fluorescence (FL2-A), asymmetrical gates were fitted with the autogating tool. Gates were centered near FSC ∼100 and FL2-A ∼300 and contained all events of sufficient contiguity as defined by the default autogating parameters, on average ∼95% of total. From the gated populations, we determined the mean and standard deviation of the FSC parameter. Cell cycle phase subpopulations were computed from the gated population using the Dean-Jett-Fox model without constraints. %G1 was defined as the area of the G1 model peak, divided by the combined areas of the G1 and G2/M peaks. Because the %G1 and associated parameters represent continua across experiments, it was necessary to identify model fits that did not accurately represent experimental data. This was assessed primarily by root mean square (RMS) error and ratio of mean fluorescence intensities (MFI, calculated from the FL2-A parameter) of the G2/M vs. G1 peaks. Automated unconstrained analyses that yielded extremes in these parameters, or extremes in %G1 or S-phase components of the model fit, were manually constrained by application of the median G2/G1 MFI ratio and a G1 MFI position that minimized the resulting overall RMS. All model fits were visually inspected in order to confirm the accuracy of the fit. Unquantifiable data was excluded from further analysis. Experimental data and correlations are provided in the searchable spreadsheet available as Dataset S1. Raw data files can be freely accessed at Cytobank (www.cytobank.org) and are found in the public experiments “Yeast DNA Content Project - DRUG - INCLUDED” and “Yeast DNA Content Project - DRUG -EXCLUDED”.

### Proliferation assays

Yeast strain BY4741 was grown overnight at 30°C in a 1 ml YPD starter culture, then diluted 1∶200 into fresh YPD in the presence or absence of drug. 200 µl volumes were aliquoted into clear flat-bottom 96-well sterile cell culture plates (Thermo Scientific, Nunc MicroWell Plate 167008), and the absorbance at 600 nm was measured hourly using a Tecan infinite 200Pro plate reader, after one minute of 3.5 mm orbital shaking to re-suspend cells. Plates were incubated standing at 30°C in between measurements. Absorbances were blanked post-measurement against wells containing media and DMSO alone. For combination assays, cells were treated with drug at the time of initial reseeding, at a final DMSO concentration of 1.24% throughout, aliquoted immediately into 96-well plates for reading of absorbance, and followed as described above. Growth constants were calculated using a best fit for exponential growth incorporating time points from 2 h through 6 h. For order of addition experiments, cells were reseeded at 1∶200 into fresh YPD in a culture tube, cultured standing at 30°C with hourly re-suspension for 3 h, then divided into three tubes and treated with the first drug (200 µM) or DMSO-only control, at a final DMSO concentration of 0.62% throughout. Following an additional 3 h of incubation at 30°C, the primary treated cultures were washed twice with fresh YPD at 30°C, re-suspended in the same, and further divided for treatment with the second drug, as above, resulting in nine total temporal combinations of vehicle, gemfibrozil, and fluoxetine. Growth constants were calculated as above from 0 h through 6 h.

### Drugs

The FDA-approved library was purchased from Enzo (Cat. #: BML-2841). Artemisinin was from Enzo (Cat. #: ALX-350-219), gemfibrozil from Sigma (Cat. #: G9518), while chlorpromazine (Cat. #: 101077-482), fluoxetine (Cat. #: 89160-860) and clinafloxacin (Cat. #: 89150-368) were purchased through VWR International. All drug stock solutions were in DMSO.

## Supporting Information

Figure S1 **Determining the length of G1.** *Left*, Graphs from which we determined the specific rate of cell size increase constant *k*, shown in Figure 4, from the same elutriation experiments. The natural log cell size (y-axis) is plotted against time (shown in hours, x-axis). *Right*, Graphs of the fraction of budded cells (y-axis) as a function of cell size (in fl, x-axis), from the same elutriation experiments. The data points shown were used to estimate the critical size for division we show in Figure 4A. In A, the cells were treated with DMSO, in B with rapamycin (at 0.1 µg/ml), and in C with gemfibrozil (at 50 µg/ml).
(TIF)

Table S1 **Drugs that lead to a High G1 DNA content.**
(DOCX)

Table S2 **Drugs that lead to a Low G1 DNA content.**
(DOCX)

Table S3 **Fluoxetine strongly inhibits yeast cell proliferation, but it is suppressed by gemfibrozil.**
(DOCX)

Table S4 **Gemfibrozil suppresses fluoxetine's anti-proliferative effects only if added before, but not after, fluoxetine.**
(DOCX)

Dataset S1 **Searchable spreadsheet of all the primary data, arranged in different worksheets.**
(XLSX)
