# Supplementary figures and images for: Systematic Analysis of Cell Cycle Effects of Common Drugs Leads to the Discovery of a Suppressive Interaction between Gemfibrozil and Fluoxetine
Source: PLoS One. 2012 May 2;7(5):e36503. doi: 10.1371/journal.pone.0036503 (PMC3342239; doi:10.1371/journal.pone.0036503)

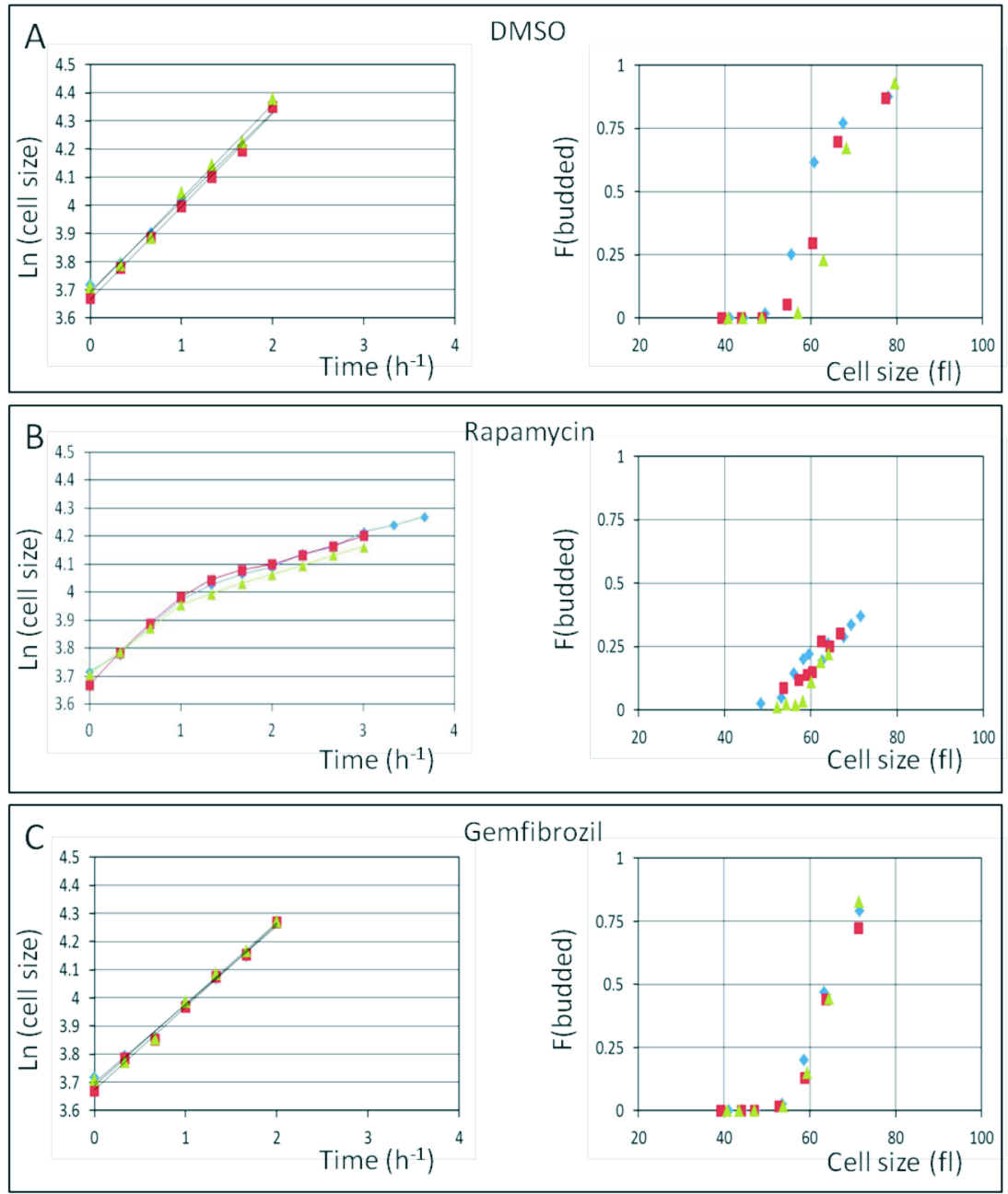

Supplement: Figure S1 — Determining the length of G1. Left, Graphs from which we determined the specific rate of cell size increase constant k, shown in Figure 4, from the same elutriation experiments. The natural log cell size (y-axis) is plotted against time (shown in hours, x-axis). Right, Graphs of the fraction of budded cells (y-axis) as a function of cell size (in fl, x-axis), from the same elutriation experiments. The data points shown were used to estimate the critical size for division we show in Figure 4A. In A, the cells were treated with DMSO, in B with rapamycin (at 0.1 µg/ml), and in C with gemfibrozil (at 50 µg/ml). (TIF) [file pone.0036503.s001.tif]
