# Supplementary material for: Systematic Analysis of Cell Cycle Effects of Common Drugs Leads to the Discovery of a Suppressive Interaction between Gemfibrozil and Fluoxetine
Source: PLoS One. 2012 May 2;7(5):e36503. doi: 10.1371/journal.pone.0036503 (PMC3342239; doi:10.1371/journal.pone.0036503)
Supplement: Table S1 — Drugs that lead to a High G1 DNA content. (DOCX) [file pone.0036503.s002.docx]

**TABLE S1. Drugs that lead to a High G1 DNA content**

| ***Drug*** | ***%G1*** | ***Use***‡ | ***Type*** |  |
| --- | --- | --- | --- | --- |
| **Auranofin**† | 95.94% | Antirheumatic | Organogold compound | |
| **Ketoconazole** | 84.05% | Antifungal | Ergosterol inhibitor | |
| **Climbazole** | 83.30% | Antifungal | Ergosterol inhibitor | |
| Oxatomide | 79.10% | Antiallergic, antiasthmatic | Histamine H1 antagonist | |
| **Rapamycin** | 78.81% | Immunosuppressant, anticancer | TOR inhibitor | |
| **Myclobutanil** | 76.56% | Antifungal | Ergosterol inhibitor | |
| Aripiprazole | 76.24% | Antipsychotic | Presynaptic dopamine agonist, postsynaptic D2 antagonist | |
| Haloperidol | 76.21% | Antipsychotic | Dopamine antagonist | |
| Flunarizine | 76.11% | Antimigraine | Calcium channel blocker | |
| **Itraconazole** | 75.14% | Antifungal | Ergosterol inhibitor | |
| Gestrinone | 73.60% | Contraceptive | Steroid |  |
| Clopidogrel | 71.92% | Platelet aggregation inhibitor | |  |
| **Dehydroepi-androsterone** | 71.86% |  | Steroid |  |
| Fluconazole | 71.14% | Antifungal | Ergosterol inhibitor | |
| **Nystatin** | 69.94% | Antifungal | Ionophore | |
| ***Drug*** | ***%G1*** | ***Use***‡ | ***Type*** |  |
| Ibudilast | 69.85% | Vasodilator | Phosphodiesterase inhibitor | |
| Lovastatin | 69.77% | Anticholesteremic | HMG-CoA reductase inhibitor | |
| Ifenprodil | 69.36% | Anticonvulsant, vasodilator | NMDA receptor inhibitor | |
| **Progesterone** | 69.33% |  | Steroid |  |
| Vatalanib | 69.08% | Antiangiogenic | Protein kinase inhibitor | |
| **Artemisinin** | 69.05% | Antimalarial |  |  |
| Nefazodone | 68.80% | Antidepressant |  |  |
| Fenretinide | 68.11% | Antineoplastic | Retinoid |  |
| **Amlodipine** | 67.31% | Antihypertensive, vasodilator | Calcium channel blocker | |
| **Gemfibrozil** | 66.69% | Antilipemic | PPARa activator | |
| **Alfacalcidol** | 65.06% | Bone density conservation | Vitamin D analog | |
| Canrenone | 64.94% |  | Steroid |  |

‡Information about the use and type for each drug was obtained from PubChem (http://pubchem.ncbi.nlm.nih.gov/).

†Drugs shown in bold were active both in *pdr5Δ, snq2Δ*, and in *PDR5^+^ SNQ2^+^* cells.
