# Supplementary material for: Systematic Analysis of Cell Cycle Effects of Common Drugs Leads to the Discovery of a Suppressive Interaction between Gemfibrozil and Fluoxetine
Source: PLoS One. 2012 May 2;7(5):e36503. doi: 10.1371/journal.pone.0036503 (PMC3342239; doi:10.1371/journal.pone.0036503)
Supplement: Table S2 — Drugs that lead to a Low G1 DNA content. (DOCX) [file pone.0036503.s003.docx]

**TABLE S2. Drugs that lead to a Low G1 DNA content**

| ***Drug*** | ***%G1*** | ***Use***‡ | ***Type*** |  |  |
| --- | --- | --- | --- | --- | --- |
| **Fluoxetine**† | 16.98% | Antidepressant | Serotonin uptake inhibitor | | |
| Promethazine | 20.39% | Antiallergic | Histamine H1 antagonist | | |
| **Moxifloxacin** | 21.17% | Antimicrobial | Topoisomerase inhibitor | | |
| **Clinafloxacin** | 25.38% | Antimicrobial | Topoisomerase inhibitor | | |
| **Mitomycin c** | 27.26% | Antineoplastic | Alkylating agent | |  |
| **Chlorpromazine** | 27.80% | Antipsychotic | Dopamine antagonist | | |
| Nadifloxacin | 28.13% | Antimicrobial | Topoisomerase inhibitor | | |
| **Idarubicin** | 29.10% | Antineoplastic | Intercalating agent | |  |
| Clozapine | 31.90% | Antipsychotic | GABA and Serotonin antagonist | | |
| **Gatifloxacin** | 33.52% | Antimicrobial | Topoisomerase inhibitor | | |
| Aclarubicin | 36.42% | Antineoplastic | Intercalating agent | |  |
| Sparfloxacin | 36.57% | Antimicrobial | Topoisomerase inhibitor | | |

‡Information about the use and type for each drug was obtained from PubChem (http://pubchem.ncbi.nlm.nih.gov/).

†Drugs shown in bold were active both in *pdr5Δ, snq2Δ*, and in *PDR5^+^ SNQ2^+^* cells.
