# Supplementary material for: Systematic Analysis of Cell Cycle Effects of Common Drugs Leads to the Discovery of a Suppressive Interaction between Gemfibrozil and Fluoxetine
Source: PLoS One. 2012 May 2;7(5):e36503. doi: 10.1371/journal.pone.0036503 (PMC3342239; doi:10.1371/journal.pone.0036503)
Supplement: Table S3 — Fluoxetine strongly inhibits yeast cell proliferation, but it is suppressed by gemfibrozil. (DOCX) [file pone.0036503.s004.docx]

**TABLE S3. Fluoxetine strongly inhibits yeast cell proliferation, but it is suppressed by gemfibrozil.**

|  | **[Gemfibrozil]** | | | |
| --- | --- | --- | --- | --- |
| **[Fluoxetine]** | **0 μM** | **50 μM** | **100 μM** | **200 μM** |
| **0 μM** | 0.41±0.03* | 0.43±0.01 | 0.45±0.01 | 0.40±0.01 |
| **50 μM** | 0.41±0.02 | 0.43±0.01 | 0.45±0.00 | 0.42±0.02 |
| **100 μM** | 0.39±0.02 | 0.45±0.01 | 0.47±0.00 | 0.43±0.01 |
| **200 μM** | 0.03±0.01 | 0.46±0.02 | 0.44±0.03 | 0.42±0.01 |

*The specific growth rate constant (*k*) of each drug combination is shown. These values were used to generate the graph in Figure 5A. Errors represent the standard deviation of nine replicates of the 0 µM/0 µM control, and ranges of two replicates of all others.
