# Supplementary material for: Systematic Analysis of Cell Cycle Effects of Common Drugs Leads to the Discovery of a Suppressive Interaction between Gemfibrozil and Fluoxetine
Source: PLoS One. 2012 May 2;7(5):e36503. doi: 10.1371/journal.pone.0036503 (PMC3342239; doi:10.1371/journal.pone.0036503)
Supplement: Table S4 — Gemfibrozil suppresses fluoxetine's anti-proliferative effects only if added before, but not after, fluoxetine. (DOCX) [file pone.0036503.s005.docx]

**TABLE S4. Gemfibrozil suppresses fluoxetine’s anti-proliferative effects only if added before, but not after, fluoxetine.**

|  | **2^nd^ treatment** | | |
| --- | --- | --- | --- |
| **1^st^ treatment** | **DMSO** | **Gemfibrozil** | **Fluoxetine** |
| **DMSO** | 0.470±0.001* | 0.400±0.002 | 0.108±0.005 |
| **Gemfibrozil** | 0.475±0.006 | 0.402±0.001 | 0.277±0.004 |
| **Fluoxetine** | 0.287±0.001 | 0.239±0.001 | -0.008±0.001 |

*The specific growth rate constant (*k*) of each treatment combination is shown. These values were used to generate the graph in Figure 5B. Errors represent the ranges of two replicates for all samples.
